# Evaluating Retinal Disease Diagnosis with an Interpretable Lightweight CNN Model Resistant to Adversarial Attacks

**DOI:** 10.3390/jimaging9100219

**Published:** 2023-10-11

**Authors:** Mohan Bhandari, Tej Bahadur Shahi, Arjun Neupane

**Affiliations:** 1 Department of Science and Technology, Samriddhi College, Bhaktapur 44800, Nepal; mail2mohanbhandari@gmail.com; 2 School of Engineering and Technology, Central Queensland University, Norman Gardens, Rockhampton, QLD 4701, Australia; a.neupane@cqu.edu.au; 3 Central Department of Computer Science and IT, Tribhuvan University, Kathmandu 44600, Nepal

**Keywords:** adversarial attacks, deep learning, health informatics, lightweight CNN, retinal image classification

## Abstract

Optical Coherence Tomography (OCT) is an imperative symptomatic tool empowering the diagnosis of retinal diseases and anomalies. The manual decision towards those anomalies by specialists is the norm, but its labor-intensive nature calls for more proficient strategies. Consequently, the study recommends employing a Convolutional Neural Network (CNN) for the classification of OCT images derived from the OCT dataset into distinct categories, including Choroidal NeoVascularization (CNV), Diabetic Macular Edema (DME), Drusen, and Normal. The average k-fold (k = 10) training accuracy, test accuracy, validation accuracy, training loss, test loss, and validation loss values of the proposed model are 96.33%, 94.29%, 94.12%, 0.1073, 0.2002, and 0.1927, respectively. Fast Gradient Sign Method (FGSM) is employed to introduce non-random noise aligned with the cost function’s data gradient, with varying epsilon values scaling the noise, and the model correctly handles all noise levels below 0.1 epsilon. Explainable AI algorithms: Local Interpretable Model-Agnostic Explanations (LIME) and SHapley Additive exPlanations (SHAP) are utilized to provide human interpretable explanations approximating the behaviour of the model within the region of a particular retinal image. Additionally, two supplementary datasets, namely, COVID-19 and Kidney Stone, are assimilated to enhance the model’s robustness and versatility, resulting in a level of precision comparable to state-of-the-art methodologies. Incorporating a lightweight CNN model with 983,716 parameters, $2.37\times 10^{8}$ floating point operations per second (FLOPs) and leveraging explainable AI strategies, this study contributes to efficient OCT-based diagnosis, underscores its potential in advancing medical diagnostics, and offers assistance in the Internet-of-Medical-Things.

## 1. Introduction

The retina, situated at the posterior aspect of the ocular globe, comprises photoreceptor cells adept at transducing luminous stimuli into intricate electrical signals, subsequently dispatched to the cerebral cortex via the optic nerve. This intricate process serves as the foundation for human visual perception, wherein the brain deciphers these electrical transmissions as coherent visual representations. Retinal diseases can seriously affect vision and in some cases, can lead to permanent blindness [1] which is a big problem for the general health of the public. Getting a prompt and accurate diagnosis with the help of automated tools is a great assist for medical specialists in making wise medical decisions. The advancement of digital medical imaging has brought about a significant change in ophthalmology as it has introduced effective technologies that help in the detection of such diseases. By improving early detection through image analysis and identifying minuscule anomalies, Artificial Intelligence (AI) has considerably coped with retinal diseases. AI has also enhanced treatment planning by analyzing patient data and enabling tailored care. Additionally, AI-driven systems help track the development of diseases, resulting in therapies that are more successful [2].

Different Machine Learning (ML) and Convolutional Neural Networks (CNNs) are efficient at analyzing images and are particularly incredible at recognizing complex patterns in medical images [3]. Their ability to diagnose complicated retinal diseases is efficient without a doubt, but in medical practice, using CNNs depends not only on how well they can diagnose the issues but also on how useful they are in places with limited computational resources. Not only CNN, but different variants of CNN like ResNet [4], VGG [5] and more have produced good accuracies statistically. These CNNs and their variants have a very high number of training parameters, and many layers which make it time-consuming in real-time predictions [6] and integration with the Internet-of-Medical-Things (IoMT) [7].

As AI technology advances, it has become essential to not only achieve better diagnostic abilities but also to understand how these AI systems make predictions and decisions [8,9,10]. As these models can be hard to understand because of their statistical nature making them black boxes [11], the addition of Explainable Artificial Intelligence (XAI) into these models can solve the problem. The combination of small and efficient CNN models in IoMT devices with XAI, as a bio-marker, helps retinal disease diagnosis to be more accurate and more accessible for medical experts, practitioners, and even ordinary people.

To resolve all of these issues, this study aims to achieve the following three key objectives:

1. To develop an efficient CNN model with minimal parameters for detecting retinal abnormalities such as CNV, DME, and Drusen using OCT datasets.
2. To incorporate Explainable Artificial Intelligence (XAI) methodologies such as Local Interpretable Model-agnostic Explanations (LIME) and Shapley additive explanations (SHAP) into the realm of clinical interpretation, with the aim of comprehending the prediction by the Convolutional Neural Network (CNN) model.
3. To generalize the model’s reliability and applicability; two new additional datasets were trained and evaluated for the model.

## 2. Related Works

Researchers in health informatics are leveraging the predictive power of Deep Learning (DL) to address the automated diagnosis of various diseases such as COVID-19 [12], monkeypox [13], kidney stone [14] and so on. Here, we summarise the recent DL methods that have been employed for retinal disease diagnosis using various image modalities. These methods can be categorized into two broad classes: pre-trained DL models (that leverage the transfer learning strategies) and custom-designed CNN (which needs training from scratch).

Subramanian et al. [15] utilised four CNN models such as VGG16, DenseNet-201, Inception-V3, and Xception, to classify seven different retinal diseases. Moreover, Bayesian optimization was employed to fine-tune the hyperparameters of these CNN models, coupled with image augmentation techniques to enhance their ability to generalize. The use of DenseNet-201 in classifying retinal diseases on the Retinal OCT Image dataset resulted in an accuracy exceeding 99%, demonstrating superior performance compared to alternative methods. Puneet et al. [16] implemented the combination of Attention and Transfer Learning approaches into a DCNN for categorizing retinal diseases such as CNV, DME, and Drusen using OCT images. Their proposal achieved notable results, attaining accuracies of 97.79% during training and 95.6% during testing. Kayadibi et al. [17] implemented a hybrid fine-tuned CNN for retinal disease classification from OCT images. They utilized PCA to reduce the feature size and enhance the performance. The benchmarking outcomes for two OCT datasets demonstrated a high level of promise in terms of accuracy. Specifically, the UCSD dataset yielded an impressive accuracy of 99.70% according to Kermany et al.’s study  [18], while the Duke dataset achieved a perfect 100% accuracy as reported by Srinivasan et al. [19]. In their research, Kim and colleagues [20] harnessed a variety of Convolutional Neural Networks (CNNs) like VGG16, ResNet50, DenseNet121, and Inception-v3 as feature extractors. Subsequently, they employed these features to develop binary OCT image classification models. A binary classifier model is developed for each category (CNV, DME, Drusen and Normal) and the VGG-16-based model for CNV vs. other classes achieved 98.6% accuracy. They achieve high accuracy using the pre-trained DL models. However, their proposal needs the training of individual models for each class which incurs high computational complexity. A pre-trained VGG-16 network was implemented by Li et al. [21] for retinal image classification on OCT images. They validated the model’s performance on 1000 independent OCT images. Their work revealed that the transfer learning with the VGG-16 model has a promising accuracy of 98.6%, sensitivity of 97.8%, and specificity of 00.4%. With such commendable performance of the model, deep learning can automate the diagnosis of retinal disease. Li et al. [22] adopted the ensemble models for retinal disease classification using OCT images. They trained four DL- models based on improved ResNet50 to build the ensemble and achieved the highest accuracy of 96.3%, sensitivity of 96.6%, and specificity of 98.7%. However, the ResNet50 model is itself the heavyweight model.

In addition to employing pre-trained deep learning models, only a limited number of researchers have created custom CNNs for the classification of retinal images. For example, a deep CNN with six convolution blocks (including the Relu, batch normalization, and pooling operation) was implemented by Sujina et al. [23]. Their proposal achieved a promising accuracy of 99.69% with a low misclassification rate. However, the generalisability of the CNN on additional datasets is not reported. Altan et al. [24] implemented a deep learning architecture to detect the macular edema on OCT images and reported an accuracy of 99.20%.

Hybrid deep learning models for retinal image classification have also been proposed recently. For instance, a hybrid deep learning model for OCT image classification was implemented by Khan et al. [25]. They extracted retinal features from OCT images using three pre-trained deep learning models (DenseNet121, InceptionV3, and ResNet50), and ant colony optimization was used for best feature selection. Finally, the SVM and KNN were employed for classification. Their proposal achieved high performance on OCT image classification. However, the approach is not applicable to end-to-end training of the model.

## 3. Materials and Methods

**Figure 1:**
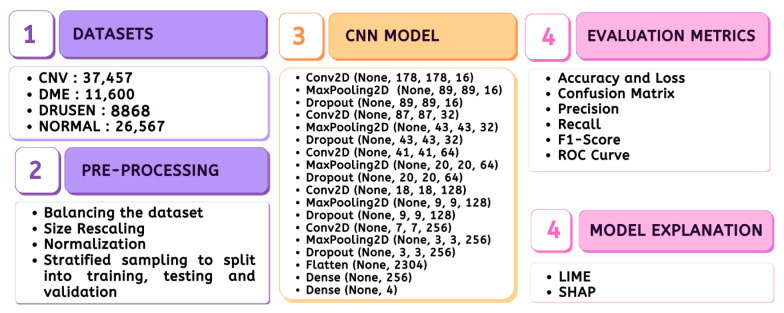
Methodology.

The entire material and methods adopted in the study are depicted in Figure 1, which includes the stages ranging from data preparation to model evaluation.

### 3.1. Dataset

#### 3.1.1. Disease Description

**Figure 2:**
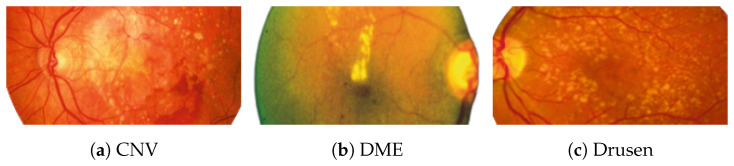
Representative images for diseases.

Figure 2 [26] shows the different diseases considered in this study. CNV, depicted in Figure 2a, arises from the emergence of fresh blood vessels in proximity to the choroid. CNV is caused by flaws in the innermost section of the choroid known as Bruch’s membrane, along with conditions like severe nearsightedness and heightened vascular endothelial growth. DME, Figure 2b, primarily affects individuals with diabetes. It leads to vision distortion as fluid accumulates in the macula. This accumulation impairs cone cells’ light-sensing abilities, causing blurred vision. DME arises from the expansion of blood vessels at the posterior region. In Figure 2c, we can observe Drusen, a condition primarily linked to the aging process. It involves the accumulation of yellow extracellular particles between the Bruch’s membrane and the retinal pigment in the eye. Drusen has the potential to hinder the transport system, which could lead to a deprivation of oxygen for the cone cells responsible for colour vision, ultimately resulting in their deterioration.

#### 3.1.2. Dataset Description

**Figure 3:**
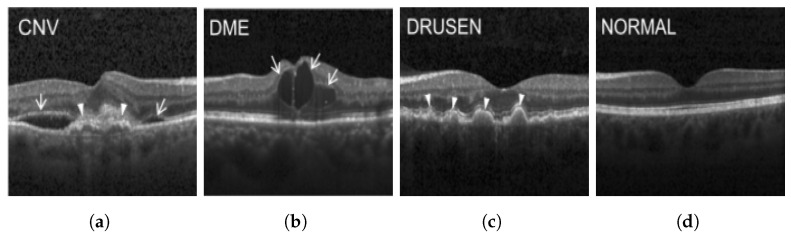
Illustrative examples from the retinal image dataset are presented. Figure (**a**) showcasing Choroidal NeoVascularization, characterized by the presence of neovascular membranes (indicated by white arrowheads) along with associated sub-retinal fluid (marked by arrows). Figure (**b**) illustrates Diabetic Macular Edema, which manifests as intra-retinal fluid associated with retinal thickening (denoted by arrows). Figure (**c**) displays multiple instances of drusen (highlighted by arrowheads), while Figure (**d**) illustrates a normal, pristine retina with an undisturbed foveal structure and no signs of retinal fluid or edema.

The publicly accessible dataset [27] encompasses detailed cross-sectional images of living patients’ retinas, which have been classified into four distinct categories: Normal, CNV, Drusen, and DME. These categories are visually represented in Figure 3. The dataset comprises a grand total of 84,492 images, distributed as follows: CNV contains 37,457 images, Normal contains 26,567 images, DME includes 11,600 images, and Drusen encompasses 8868 images.

### 3.2. Dataset Pre-Processing

The originally imbalanced dataset was transformed into a balanced one, where each category contained exactly 8868 images. In this balanced dataset, all images were resized to dimensions of 180 × 180 and then normalized to fall within the range [0, 1]. To ensure representative sampling, a stratified approach was employed, allocating 80% of the data for training, and the remaining 20% for testing, with half of the testing data reserved for validation.

### 3.3. CNN Model

**Figure 4:**
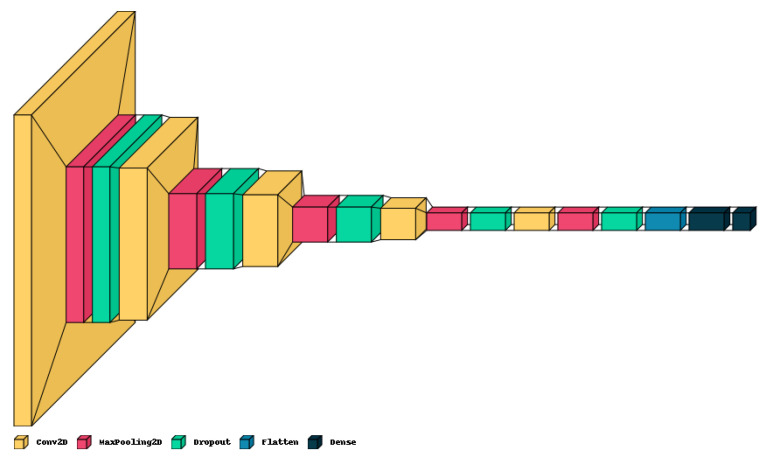
Graphical visualization of the proposed model.

The proposed lightweight model reported in Table 1 provides a concise overview of the CNN architecture and Figure 4 shows the graphical result. The model holds only five convolution layers to perform convolution operations on the input image, with increasing filter depths (16, 32, 64, 128, 256) to capture hierarchical features. Each convolution layer is followed by max-pooling layers to down-sample the feature maps, aiding in information compression. Dropout layers help mitigate over-fitting by randomly deactivating neurons during training with values of 0.2 on each. The final dense layers (256, 4) process flattened features for classification, culminating in four output classes. The model contains around 983,716 total trainable parameters, contributing to its complexity and predictive capacity.

### 3.4. Implementation Setup

The CNN model and XAI algorithms in this study were implemented using Python 3.10.12, along with Keras version 2.13.1 and TensorFlow version 2.13.0. The computational resources employed for this research included the runtime environment provided by Google Colab, supported by a robust NVIDIA K80 GPU with an impressive 12 GB of RAM. To assess the performance of the CT dataset, a cross-validation technique with a designated value of K = 10 [28] was employed, which entailed distinct and random allocations for both training and testing subsets. For the purpose of regularization, an early stopping strategy was employed, which relied on monitoring the validation loss for 10 consecutive epochs.

### 3.5. Performance Evaluation Metrics

#### 3.5.1. Accuracy

The performance of classification models is typically evaluated using the metric of accuracy. Out of all the examples in a dataset, it calculates the percentage of accurately predicted instances. Mathematically, accuracy (Acc) is calculated as Equation (1).

(1) $$Acc=\frac{\mathrm{Number}\;\mathrm{of}\;\mathrm{Correct}\;\mathrm{Predictions}}{\mathrm{Total}\;\mathrm{Number}\;\mathrm{of}\;\mathrm{Predictions}}$$

#### 3.5.2. Precision

Precision is a performance measure that assesses the correctness of a model’s positive predictions. It is determined by the ratio of true positive predictions (TP) to the sum of true positive and false positive predictions (TP+FP).

#### 3.5.3. Recall

Recall evaluates a capacity to accurately identify every positive occurrence present in the dataset. It is described as the proportion of genuine positives (positives that were correctly detected) to all real positives.

#### 3.5.4. F1-Score

The F1 Score is a classification task evaluation metric that balances precision and recall. It is calculated as the harmonic mean of precision and recall, offering a single measure of model performance that takes both false positives and false negatives into account.

#### 3.5.5. ROC Curve

The Receiver Operating Characteristic (ROC) curve is a graph that shows how well a model works. It shows the trade-off between two things: how often the model correctly says “yes” when it should (Sensitivity), and how often it incorrectly says “yes” when it shouldn’t (1-Specificity). This graph helps us see how good the model is at telling things apart in different settings. To make the ROC curve, we draw a graph of the True Positive Rate (TPR) against the False Positive Rate (FPR) for different settings. The area under the ROC curve (AUC-ROC) gives us a single number that tells us how well the model is doing.

#### 3.5.6. FLOPS

Algorithm 1 calculates the Floating Point Operations (FLOPs) for the CNN model [29]. It defines two functions, CalculateCNNLayerFLOPs, and CalculateDenseLayerFLOPs, to compute FLOPs for Conv2D and Dense layers, respectively, based on their parameters. The CalculateTotalFLOPs function iterates through the model’s layers, identifying Conv2D and Dense layers, and accumulates their respective FLOPs. This provides an estimate of the total computational complexity of the CNN model. The algorithm is valuable for assessing the computational efficiency of the CNN in terms of the number of operations needed for inference.
**Algorithm 1** Calculate FLOPs for CNN Model [29]1:**function** CalculateCNNLayerFLOPs(layer)2:      **Input:** CNN layer3:      **Output:** FLOPs for the layer4:      **return** 2×layer.filters×layer.kernel_size[0]×layer.kernel_size[1]×layer.input_shape[−1]×layer.output_shape[1]×layer.output_shape[2]5:**end function**6:**function** CalculateDenseLayerFLOPs(layer)7:      **Input:** Dense layer8:      **Output:** FLOPs for the layer9:      **return** 2×layer.input_shape[−1]×layer.output_shape[−1]10:**end function**11:**function** CalculateTotalFLOPs(model)12:      **Input:** CNN model13:      **Output:** Total FLOPs for the model14:      total_flops ← 015:      **for** layer in model.layers **do**16:          **if** layer is Conv2D **then**17:                total_flops +=CalculateCNNLayerFLOPs(layer)18:          **else if** layer is Dense **then**19:                total_flops +=CalculateDenseLayerFLOPs(layer)20:          **end if**21:      **end for**22:      **return** total_flops23:**end function**

#### 3.5.7. Explainable AI

Although there are certain challenges associated with XAI, such as its sensitivity to individual cases, the trade-off involving complexity, and the assumption of highly interdependent features, XAI delves into the visual computational approach of Deep Learning models. Consequently, the study incorporates the use of LIME and SHAP.

1. LIME
  In the pursuit of enhancing the transparency and interpretability of modern machine learning models, LIME has emerged as a powerful technique. LIME addresses the challenge of understanding complex black-box models’ predictions by approximating their behaviour through locally interpretable models. This approach allows us to shed light on how specific features influence predictions, especially in contexts involving intricate data types such as images.
  LIME operates by selecting a target instance *x*, model *f* and generating perturbed instances $x_{i}^{\prime }$ in its vicinity. The model’s predictions f(x) and $f(x_{i}^{\prime })$ are obtained, and interpretable features $z_{i}$ are extracted from the perturbed instances. An interpretable CNN model g(z), was trained using pairs $\left(z_{i},f\left(x_{i}^{\prime }\right)\right)$ to approximate the complex behavior of *f* in the local neighborhood of *x*. To analyse g(z), coefficients $\beta_{i}$ in g(z), the importance of the corresponding features $z_{i}$ in influencing the predictions were reflected. Larger absolute values of $\beta_{i}$ indicate stronger influences [30].
2. SHAP
  For the retinal input retinal image *x* with *N* number of pixels and *f* prediction, SHAP values for each pixel in the image were calculated. The values of SHAP show the contribution of the model to define how much each pixel *i* in the retinal image *x* contributes and is calculated using Equation (2).
  (2) $$\phi_{i}\left(x\right)=\sum_{S\subseteq \{1,2,\ldots ,N\}\setminus \{i\}}\frac{|S|!(N-|S|-1)!}{N!}\left[f\left(x_{S}\cup \left\{i\right\}\right)-f\left(x_{S}\right)\right]$$
  *S* represents a subset of pixels excluding pixel *i*, $x_{S}$ is the image with the pixels in subset *S* unchanged, $f(x_{S}\cup \left\{i\right\})$ is the model’s prediction when pixel *i* is included in the subset *S* and $f(x_{S})$ is the model’s prediction when pixel *i* is excluded from the subset *S* [31].
  These SHAP values provide insights into the contribution of each pixel to the model’s prediction. Positive SHAP values indicate that a pixel’s presence positively influenced the forecast, while negative values suggest the opposite.

## 4. Results

### 4.1. Statistical Results

We analyzed classic statistical validation measures, which included the model’s performance in terms of error and correctness throughout training, as well as across the validation and test datasets. Furthermore, we incorporated precision, F1-score, recall, confusion matrix, and k-fold validation into our evaluation.

Among ten different folds, 10^*th*^ fold, stopped early in 15^*th*^ epoch, holding the lowest accuracy, and the same is considered to plot the evaluation metrics. The training process spanned 20 epochs, with each fold configured to terminate early if the validation loss persisted for five consecutive epochs.

**Figure 5:**
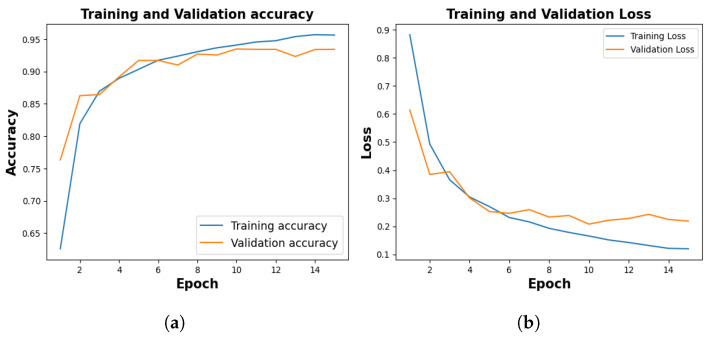
Training and Validation Result. (**a**) represents training and validation accuracy (**b**) shows training and validation loss.

Figure 5, shows the training and validation accuracy of 10^*th*^ fold where training phase yielded 95.64% and a loss of 0.1201, and the validation accuracy stood at 94.12% with a corresponding loss of 0.2185.

**Figure 6:**
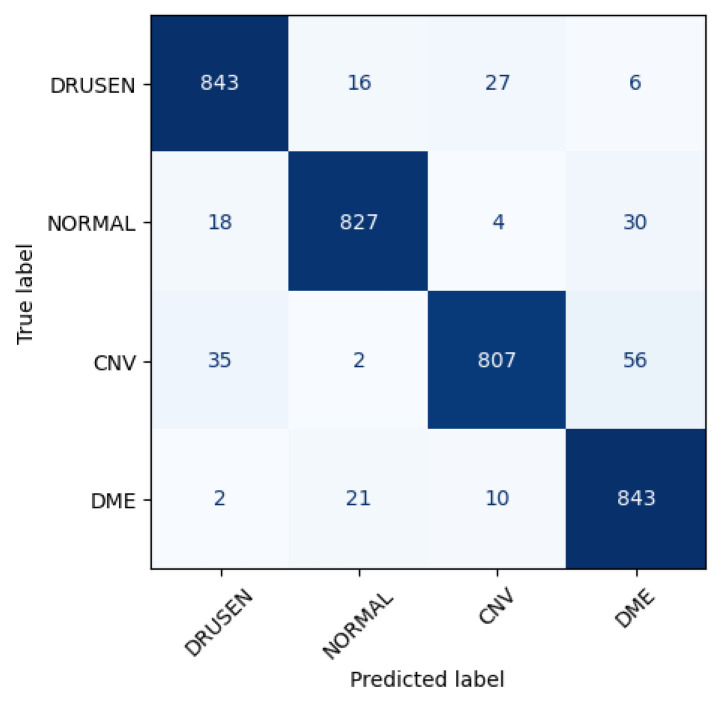
Class-wise performance of CNN model.

Figure 6 shows the performance matrix of 10^*th*^ fold. Among 892 Drusen samples, 843 were accurately predicted. Only 52 normal samples were misclassified, out of which 18 were predicted as Drusen, 4 were predicted as CNV, and 30 were predicted as DME. Out of 900 samples, a total of 807 samples were predicted correctly for the CNV class. Considering 876 samples, 843 samples were predicted correctly for DME.

Following 10 folds, the model demonstrated an average training accuracy of 96.33%, a validation accuracy of 94.12%, a training loss of 0.1073, and a validation loss of 0.1927. The average testing accuracy and testing loss stood at 94.29% and 0.2002 respectively (Table 2).

Table 3 shows the classification report where “Drusen” and “Normal” show high Precision (0.94–0.95) indicating accurate positive predictions and DME shows high Recall (0.96), capturing most positives. F1-Score ranges from 0.94 to 0.95 showing the harmony between Precision and Recall.

**Figure 7:**
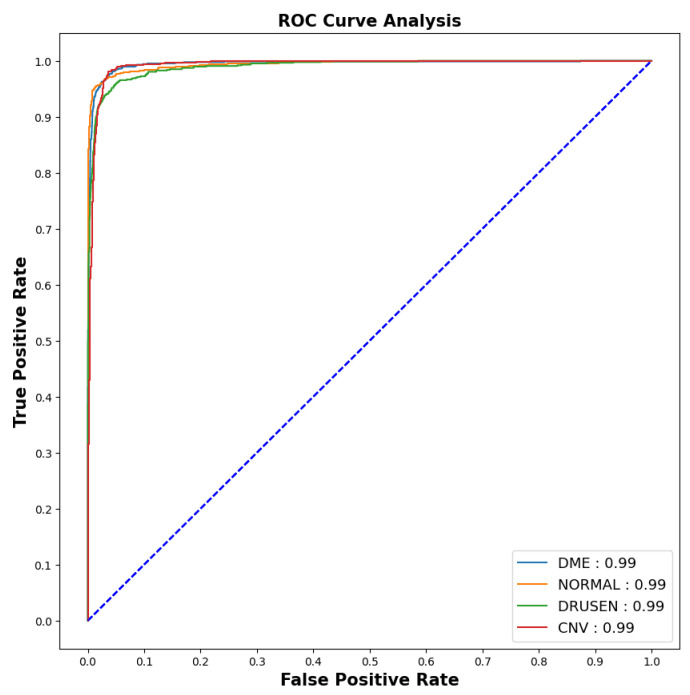
ROC-AUC curve.

As the imbalanced datasets were made balanced, the ROC curve (for 10^*th*^ fold) was plotted as shown in Figure 7 to calculate the area under the curve and evaluate the model performance.

The model showed outstanding performance in distinguishing between positive and negative categories, as proven by its impressive AUC score of 0.99 in all areas. The ROC curve, which depends on TPR and FPR, displayed the model’s predictions on the test dataset having an exceptionally high TPR, covering the entire range of AUC values. This demonstrates the model’s exceptional effectiveness.

Table 4 presents sensitivity and specificity values of four classes in 10^*th*^ fold. DME and Normal classes exhibit high sensitivity (>0.98), indicating accurate detection of relevant cases. Drusen has slightly lower sensitivity, while CNV has the highest specificity (>0.97), suggesting strong performance in distinguishing its class.

### 4.2. Explainable Results

#### 4.2.1. SHAP

**Figure 8:**
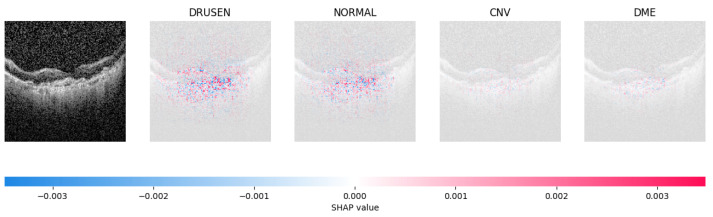
The model determined the presence of Drusen in the image by analyzing the OCT image and noting a significant concentration of red pixels (scattered in central regions) in the explanatory image (second in a row), which is located in the second column.

**Figure 9:**
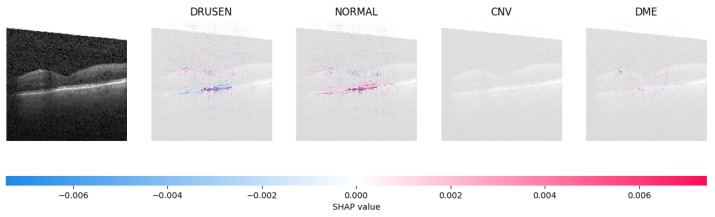
The model’s examination of the OCT image revealed a significant number of red pixels in the explanatory image (third in a row) to suggest the presence of a healthy eye.

**Figure 10:**
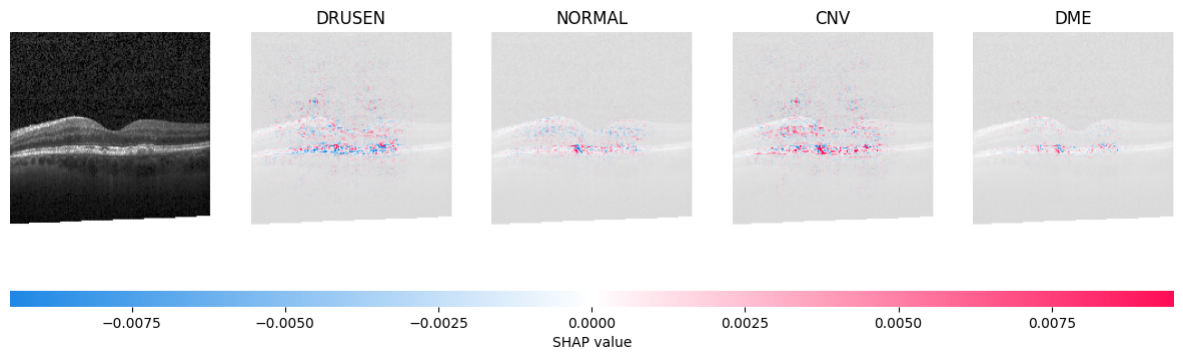
The model determined that the OCT image exhibited indications of CNV, which is a retinal disorder, due to the significant abundance of red pixels in the third explanatory image located in the fourth column.

**Figure 11:**
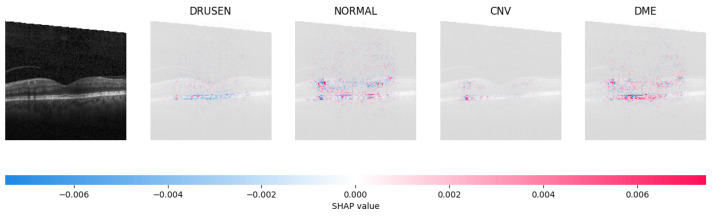
The model determined that the OCT image indicated the presence of DME due to the significant number of red pixels observed in the fifth column of the fourth explanatory image.

Because it is hard to understand how the CNN model predicted the output, XAI techniques are used to explain it [32]. The testing images are on the left, and each explanation has a transparent grey background (see Figure 8, Figure 9, Figure 10 and Figure 11).

The red pixels in the first explanation image (refer to Figure 8) increase the probability of predicting a Drusen. In the second explanation, the model somehow attempts to indicate that the image is normal, but the red pixels’ concentration in the first explanation is higher. Third, and fourth explanation images do not contain any red or blue pixels, so the probability of classifying the input image as CNV and DME is low.

In Figure 9, the presence of concentrated red pixels suggests that the image is normal. Conversely, in Figure 10 and Figure 11, the absence of concentrated red pixels indicates that they correspond to CNV and DME, respectively.

#### 4.2.2. LIME

LIME is employed to extract the features and perturbations from the training dataset. These perturbations are randomly generated from a standardized image, and subsequent operations involving mean-centering and scaling are conducted. The simple linear iterative clustering (SLIC) [33] is used to compute the initial three characteristics which delineate the most influential boundaries and incorporate them into the image. The second column of Table 5 displays the original test images corresponding to each category. The segmented image segment in the third column of Table 5 represents the segmentation obtained through LIME. As illustrated in Table 5, LIME furnishes visual explanations of the model’s decision-making process, spotlighting the image regions that make a significant contribution to a specific class prediction.

## 5. FLOPS Calculation

The proposed model’s Floating-point Operations (FLOPs) were determined by considering all arithmetic operations involving floating-point values, such as addition, subtraction, division, multiplication, and any other relevant operations. The model executed a total of $2.37\times 10^{8}$ operations, and this calculation was accomplished using Algorithm 1.

## 6. Generation of Fast Gradient Sign Method for Adversarial Examples

To evaluate the model’s robustness, we conducted tests using adversarial examples. We computed the gradient of the loss function with respect to the input images to capture subtle variations. We introduced epsilon as a hyperparameter to quantify the perturbation’s intensity, which was generated by taking the sign of the gradient and adjusting its magnitude. Subsequently, we incorporated this perturbation into the image and forwarded it for prediction. Our findings indicate that the model exhibited resilience up to an epsilon value of 0.1, as illustrated in Table 6 for DRUSEN and CNV, whereas all categories remained stable below the threshold of 0.1.

## 7. Generalizability Investigation

To see if the proposed model could be used to diagnose other common datasets with the same number of categories, the model was trained under the same constraints as before and the results were analysed for two additional datasets, COVID-19 and Kidney Stone.

### 7.1. COVID-19

**Figure 12:**
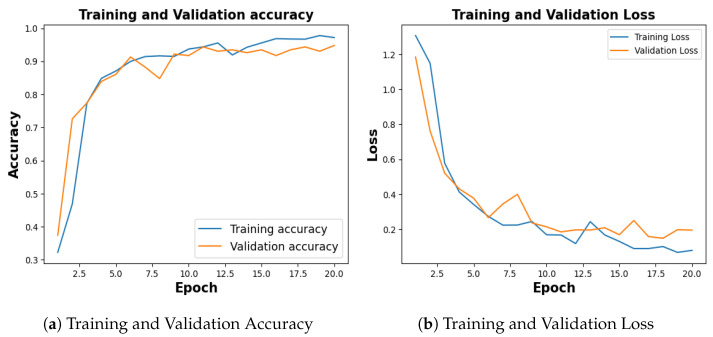
Training and Validation Results for COVID-19.

Publicly available COVID-19 dataset [34], with 4273 pneumonia samples, 1583 normal samples, 703 tuberculosis samples, and 576 COVID-19 samples was balanced with an equal number of 576 images for each category. With a training accuracy of 97.18%, a test accuracy of 92.54%, and a validation accuracy of 94.78% as shown in Figure 12a, the model achieved a training loss of 0.0804, test loss of 0.2180 and validation loss of 0.1960 as shown in Figure 12b.

**Figure 13:**
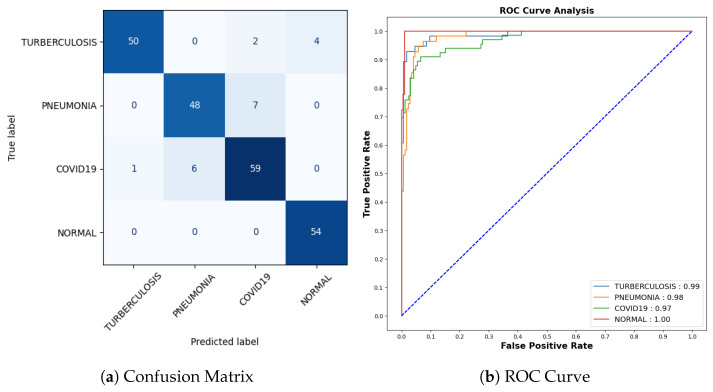
Confusion Matrix and ROC for COVID-19.

Figure 13a represents the confusion matrix. Here, six instances of Tuberculosis samples were inaccurately predicted out of a comprehensive pool of 56 samples. Seven mispredictions were observed among pneumonia samples out of 55. Similarly, for COVID-19 cases, seven errors emerged out of 66 samples, whereas all 54 samples categorized as normal were accurately predicted. To offer a more detailed insight into the findings, it is worth noting that the AUC-ROC values for tuberculosis, pneumonia, COVID-19, and normal cases stand at 0.99, 0.98, 0.97, and 1.00, respectively, as shown in Figure 13b.

**Figure 14:**
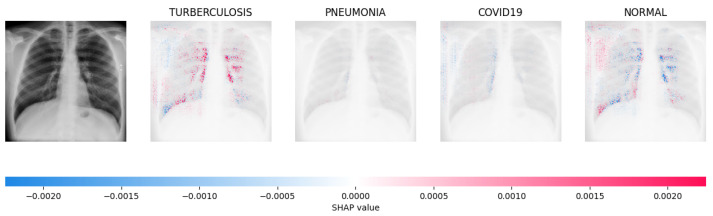
The analysis conducted by the model on the X-ray image indicated elevated concentrations of red pixels in the initial explanatory image (located in the second column). These red pixels are likely to represent regions of the image that are suggestive of tuberculosis.

**Figure 15:**
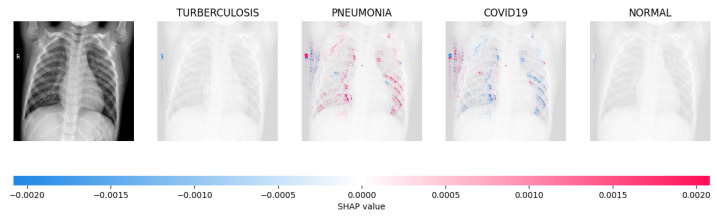
The model’s prediction is based on the presence of a substantial number of red pixels in the second explanatory image, situated in the third column, suggesting that the X-ray image depicts pneumonia.

**Figure 16:**
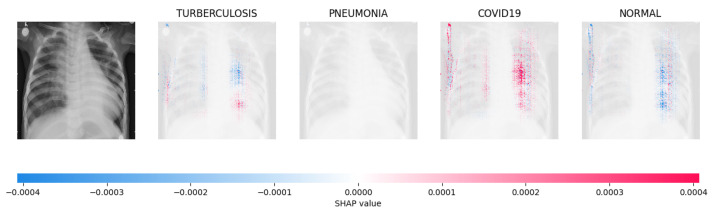
The model’s forecast of pneumonia was substantiated by the elevated density of red pixels in the third explanatory image.

**Figure 17:**
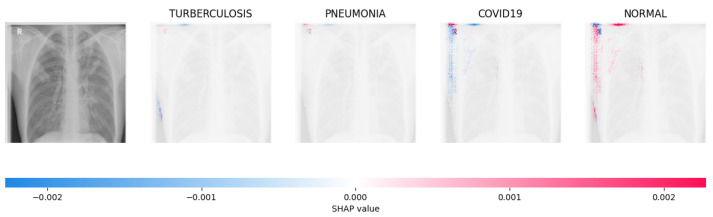
The model determined that the X-ray image was classified as “Normal” because there was a notable concentration of red pixels in the fourth explanatory image.

Figure 14, Figure 15, Figure 16 and Figure 17 show the SHAP explanation of tuberculosis, pneumonia, COVID-19, and Normal samples respectively.

**Figure 18:**
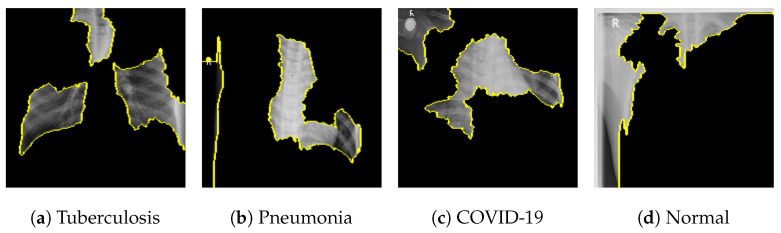
Results for LIME segmentation for Figure 14, Figure 15, Figure 16, and Figure 17 respectively.

Figure 18 shows the LIME segmented results for individual categories of the COVID-19 dataset. The segmented results highlight the infected regions in respective images.

The proposed model shows better results in comparison with other SOTA approaches and the results are tabulated in Table 7 showing training accuracy of 97.18% and number of parameters (0.983 million).

### 7.2. Kidney Stone

**Figure 19:**
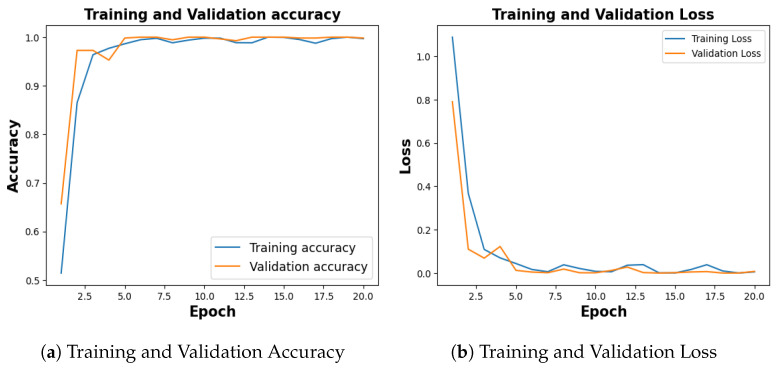
Training and Validation Result for Kidney Stone.

The publicly available Kidney Stone dataset [14], with 5077 normal samples, 3709 cyst samples, 2283 tumor samples and 1377 stone samples was balanced with an equal number of 1377 images for each category. With a training accuracy of 99.70%, a test accuracy of 99.64%, and a validation accuracy of 99.82% as shown in Figure 19a, the model achieved a training loss of 0.0056, test loss of 0.0345 and validation loss of 0.0078 as shown in Figure 19b.

**Figure 20:**
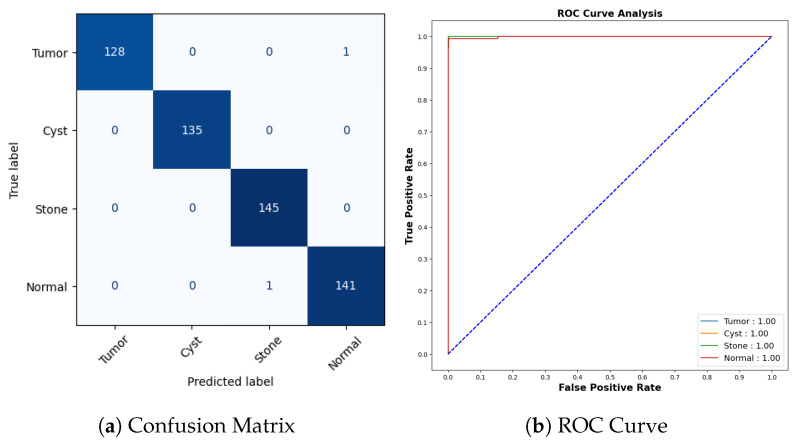
Confusion Matrix and ROC for COVID-19.

Figure 20a represents the confusion matrix for the kidney stone dataset. All cyst and stone samples were correctly classified. One tumor sample was misclassified as normal, and one normal sample was misclassified as stone. The AUCROC values for all categories are 1.00, which indicates that the model has perfect accuracy as shown in Figure 20b.

**Figure 21:**
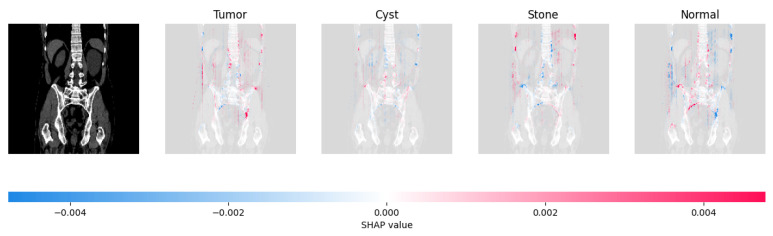
The model found that there were a lot of red pixels in the first explanation image (second column) of the CT scan. These red pixels are likely to be areas of the image that are indicative of a tumor.

**Figure 22:**
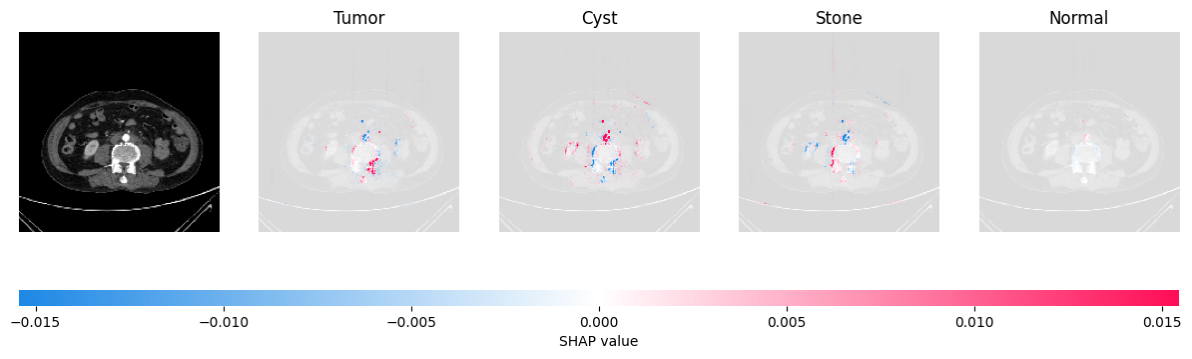
The model’s prediction that the CT image is a cyst is supported by the high concentration of red pixels in the explanation image in the third column.

**Figure 23:**
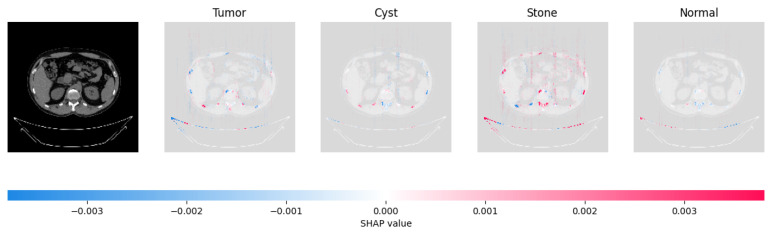
The model’s prediction of Stone was supported by the high concentration of red pixels in the third explanation image.

**Figure 24:**
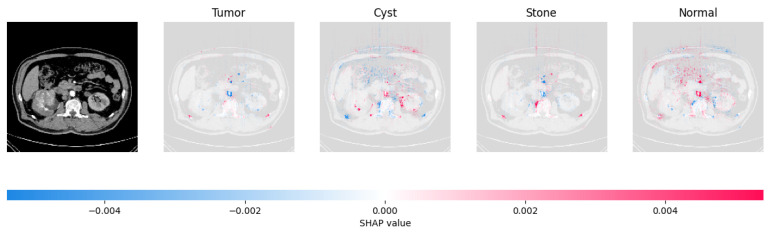
The model found that the CT image is predicted as Normal as the high concentration of red pixels is located in the fourth explanation image.

Figure 21, Figure 22, Figure 23, and Figure 24 show the SHAP explanation of tumor, cyst, and Normal samples respectively.

**Figure 25:**
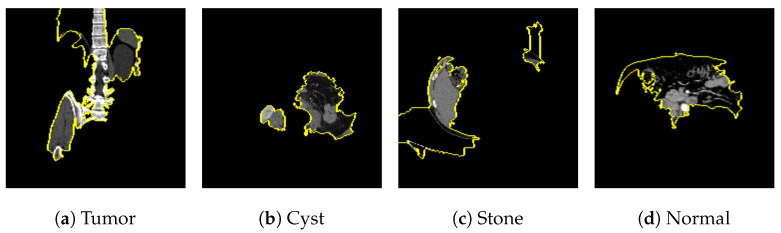
Results for LIME segmentation for Figure 21, Figure 22, Figure 23, and Figure 24, respectively.

Figure 25 shows the LIME segmented results for individual categories of the kidney stone dataset. The segmented results highlight the infected regions in respective images.

The proposed model shows the competitive results in comparison with other state-of-the-art methods as tabulated in Table 8 in terms of training accuracy (99.70%) and number of parameters (0.983 million).

## 8. Conclusions

The study presents a significant advancement in OCT-based diagnostic methodologies to address the labor-intensive nature of manual anomaly classification in retinal images. Achieving remarkable average accuracies and minimal losses across training, validation and test sets, the proposed model demonstrates its efficacy in classifying CNV, DME, Drusen, and Normal retinal conditions. The integration of XAI techniques provides interpretable insights into the model’s decision-making process. Moreover, the model’s robustness and generalizability are substantiated by its consistent performance on additional datasets related to COVID-19 and Kidney Stone conditions. With a focus on efficiency and lightweight, the model can play a significant role in IoMT devices.

With more real-time datasets, augmentation, and generative adversarial networks, other lightweight transfer learning models like MobileNet can be tested further as real-time sensations.

## Figures and Tables

**Table 1:** The proposed lightweight CNN Model Architecture. Note the “Param #.” represents the parameters involved in the given CNN.

Layer (Type)	Output Shape	Param #
Conv2D	(None, 178, 178, 16)	448
MaxPooling2D	(None, 89, 89, 16)	0
Dropout	(None, 89, 89, 16)	0
Conv2D	(None, 87, 87, 32)	4640
MaxPooling2D	(None, 43, 43, 32)	0
Dropout	(None, 43, 43, 32)	0
Conv2D	(None, 41, 41, 64)	18,496
MaxPooling2D	(None, 20, 20, 64)	0
Dropout	(None, 20, 20, 64)	0
Conv2D	(None, 18, 18, 128)	73,856
MaxPooling2D	(None, 9, 9, 128)	0
Dropout	(None, 9, 9, 128)	0
Conv2D	(None, 7, 7, 256)	295,168
MaxPooling2D	(None, 3, 3, 256)	0
Dropout	(None, 3, 3, 256)	0
Flatten	(None, 2304)	0
Dense	(None, 256)	590,080
Dense	(None, 4)	1028
Total params.: 983,716		
Trainable params.: 983,716		
Non-trainable params.: 0		

**Table 2:** Performance Metrics for Different Folds. Symbols: TA, TL, TP, TR, VA, VL, VP, VR, TeA, TeL, TeP, and TeR represent training accuracy, training loss, training precision, training recall, validation accuracy, validation loss, validation precision, validation recall, test accuracy, test loss, test precision, and test recall, respectively in percentages.

K	TA	TL	TP	TR	VA	VL	VP	VR	TeA	TeL	TeP	TeR
1	96.31	0.1011	96.49	96.13	94.11	0.1820	94.37	93.99	94.28	0.1974	94.43	94.17
2	96.22	0.1010	96.39	96.04	94.01	0.1819	94.27	93.90	94.18	0.1972	94.33	94.07
3	96.31	0.1011	96.49	96.13	94.11	0.1820	94.37	93.99	94.28	0.1974	94.43	94.17
4	96.41	0.1012	96.59	96.23	94.20	0.1822	94.46	94.09	94.37	0.1976	94.52	94.26
5	96.51	0.1013	96.69	96.33	94.30	0.1824	94.56	94.19	94.47	0.1978	94.62	94.36
6	96.31	0.1135	96.49	96.13	94.10	0.1908	94.36	93.99	94.27	0.2029	94.42	94.16
7	96.81	0.1012	96.99	96.63	94.60	0.1781	94.86	94.49	94.77	0.2015	94.92	94.66
8	96.14	0.1176	96.32	95.96	93.93	0.2160	94.19	93.82	94.10	0.2101	94.25	93.99
9	96.64	0.1151	96.82	96.46	94.43	0.2135	94.69	94.32	94.60	0.1975	94.75	94.49
10	95.64	0.1201	95.82	95.46	93.43	0.2185	93.69	93.32	93.60	0.2025	93.75	93.49
Average	96.33	0.1073	96.51	96.15	94.12	0.1927	94.38	94.01	94.29	0.2002	94.44	94.18

**Table 3:** Classification Report of 10^*th*^ fold.

Class	Precision	Recall	F1-Score
Drusen	0.94	0.95	0.94
Normal	0.95	0.94	0.95
CNV	0.95	0.90	0.92
DME	0.90	0.96	0.93
Accuracy			0.94
Macro Avg	0.94	0.94	0.94
Weighted Avg	0.94	0.94	0.94

**Table 4:** Sensitivity and Specificity of 10^*th*^ fold.

Class	Sensitivity	Specificity
DME	0.989963	0.916084
NORMAL	0.99096	0.948488
DRUSEN	0.974254	0.925115
CNV	0.966781	0.974166

**Table 5:** Interpretation of LIME results alongside input image and segmented image.

Category	OCT Image	LIME Interpretation
DRUSEN	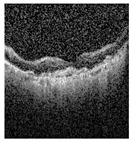	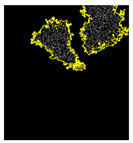
Normal	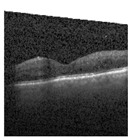	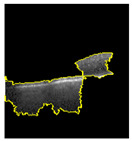
CNV	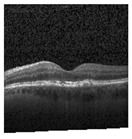	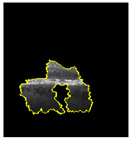
DME	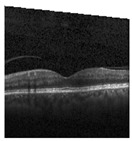	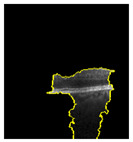

**Table 6:** Adversarial Examples.

Class	Original Image	Epsilon = 0.1	Adversarial Prediction
Drusen	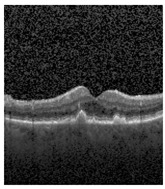	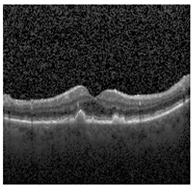	Drusen
Normal	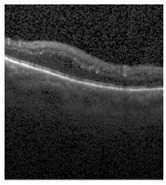	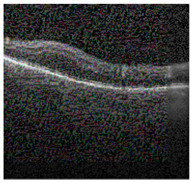	Drusen
CNV	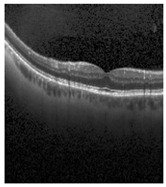	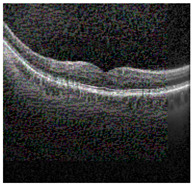	CNV
DME	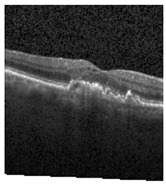	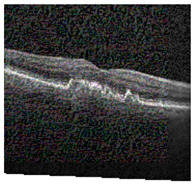	CNV

**Table 7:** Comparative Analysis of COVID-19 Result to other SOTA methods.

Ref	Algorithm	Accuracy (%)	Parameters (Millions)
[35]	CNN-based CoroNet	89.6	33.97
[36]	Custom CNN	94.53	34.73
[37]	Attention based VGG	85.43	VGG-16 = 18
			VGG-19 = 21.2
[12]	Custom CNN	95.94	3.7
Proposed	Custom CNN	97.18	0.983

**Table 8:** Comparative Analysis of the Kidney Stone dataset.

Ref	Algorithm	Accuracy (%)	Parameters (Millions)
[14]	Inception V3	61.6	22.32
	VGG16	98.2	14.74
	Resnet	73.8	23.71
	EANet	77.02	6
	Swin Transformers	99.3	4.12
	CCT	96.54	4.07
[38]	DenseNet201-Random Forest	99.44	20
[39]	VGG16NB	96.26	14.74
	DenseNet121-KNN	96.64	20
	VGG-DN-KNN	100	14.74
Proposed	Custom CNN	99.70	0.983

## Data Availability

The data used in the study is publicly accessed on 29 June 2023 https://data.mendeley.com/datasets/rscbjbr9sj/2. The COVID-19 dataset and Kidney Stone dataset are publicly available and cited with URLs in respective sections. Codes will be made available upon request.
